# Internal Carotid Artery Occlusion as a Rare Presentation of Infectious Endocarditis: A Case Report

**DOI:** 10.5811/cpcem.1489

**Published:** 2023-07-03

**Authors:** Thijs Wolf, Kayla Dewey, David Adler

**Affiliations:** University of Rochester Medical Center, Strong Memorial Hospital, Division of Emergency Medicine, Rochester, New York

**Keywords:** septic embolism, infectious endocarditis, stroke, case report

## Abstract

**Introduction:**

Internal carotid artery occlusion as a result of a septic embolism is a rare, commonly fatal, complication of mitral valve infectious endocarditis. Prompt recognition of this condition by the emergency physician may improve the chance of functional neurological survival.

**Case Report:**

A 50-year-old male presented minimally responsive with a right gaze deviation, left hemiparesis, and a score of 26 on the National Institutes of Health Stroke Scale. A point-of-care echocardiogram showed a large mitral valve vegetation, and computed tomography angiography demonstrated an internal carotid artery occlusion.

**Conclusion:**

The emergency physician should consider this potentially life-threatening condition and know the fundamental management recommendations once identified.

## INTRODUCTION

Septic emboli occur when a blood vessel is obstructed, typically as a result of a thrombus that travels from an infected source. Following an ischemic stroke, the initial ischemic insult often occurs from vascular occlusion; a second insult can occur later if the embolism results in a nidus of infection.^1^ Over the last two decades there has been a steady rise in the incidence of infectious endocarditis (IE). One study showed a 30% increase in the incidence of IE from 2000 to 2011,^2^ thought to be due to the increase in implantable cardiac devices, healthcare-associated infections, and the rise in intravenous (IV) drug use.^3^,^4^ Infectious endocarditis is known to cause ischemic stroke, but complete occlusion of the internal carotid artery is extremely rare with only a few case reports in the literature. We present a case of a middle-aged male who presented with devastating neurological symptoms in the setting of mitral valve vegetation and complete internal carotid artery occlusion.

## CASE REPORT

A 50-year-old male presented to the emergency department (ED) via emergency medical services (EMS) after being found minimally responsive in his bed at home by his roommate. He had a prior history of mitral valve endocarditis, IV heroin use, tobacco use, and atrial fibrillation on anticoagulation. The patient was last witnessed in his normal state of health the evening before his presentation to us. He had been recently hospitalized at another local hospital for methicillin-resistant *Staphylococcus aureus* bacteremia with documentation of known mitral valve vegetations, but he had left against medical advice 10 days prior. On arrival at our ED, he was ill-appearing and in acute distress with a Glasgow Coma Score of 8 (Eye 2, Verbal 2 Motor 4). The patient was afebrile, with a heart rate of 140 beats per minute with an irregular rhythm, blood pressure of 131/78 millimeters (mm) of mercury, respiratory rate of 30 breaths per minute, and pulse oximetry of 100% on a non-rebreather mask placed by EMS. The patient’s blood sugar was reported as 167 milligrams per deciliter (mg/dL) on arrival.

**Image 1:**
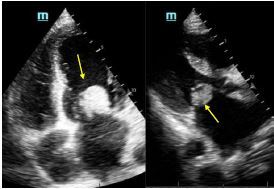
(Left) Point-of-care transthoracic echocardiogram in the apical four-chamber and (right) parasternal long-axis views demonstrating a large mitral valve vegetation (arrow).

The patient would occasionally groan but did not follow commands. Cardiac exam was notable for tachycardia with an irregular rhythm. Pupils were three mm, equal but sluggishly reactive with a right conjugate gaze deviation. The patient withdrew from pain in his right upper and lower extremities but did not localize to pain and had left-sided hemiparesis. His score on the National Institute of Health Stroke Scale was 26. During his previous hospitalization 10 days earlier the patient was documented to have a normal neurological exam. Skin exam showed pallor with scattered track marks on his upper extremities. Point-of-care transthoracic echocardiogram (TTE) (Image 1) showed a large mitral valve vegetation. Laboratory data showed a leukocytosis of 30,900/mm^3^ (reference range 4,500–11,000/mm^3^) with neutrophilic predominance, plasma lactate of 7.3 millimoles per liter (mmol/L) (0.5–2.2 mmol/L), and an acute kidney injury with a serum creatinine of 3.48 mg/dL (0.74–1.35 mg/dL).

**Image 2:**
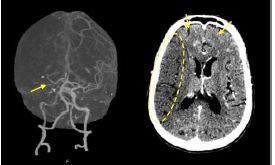
(Left) Computed tomography angiography of the head demonstrating a decrease in attenuation and caliber distal to the bifurcation (arrow) at the level of the internal carotid artery terminus with faint intermittent contrast distally that quickly dissipates. There is poor leptomeningeal collateral flow in the right hemisphere. (Right) Acute right middle cerebral artery infarction (circle) with slight effacement of the sulci and right to left shift with faint peripheral petechial hemorrhage (arrow).

A computed tomography (CT) without contrast and a CT angiography of the head (Image 2) and neck were performed demonstrating a right-sided internal carotid artery terminus occlusion with poor leptomeningeal collateral flow to the right cerebral hemisphere. Additionally, there were small areas of hemorrhagic transformation in his bilateral hemispheres, right greater than left.

The patient’s neurological state worsened, and the decision was made to proceed with rapid sequence intubation. Broad-spectrum antibiotics were also started. The patient was deemed not to be a candidate for thrombolytics or embolectomy due to the extent of vascular insult seen on his imaging. Shortly after admission, two peripheral blood cultures grew *Staphylococcus aureus* (time to positivity 6.6 hours). The patient was admitted to the neuromedical intensive care unit and ultimately died from his condition several days later.

**CPC-EM Capsule**

What do we already know about this clinical entity?

*Common risk factors for septic embolisms are intravenous drug use, infectious endocarditis, and prosthetic cardiac valves.*

What makes this presentation of disease reportable?

*Although septic emboli can occasionally cause large vessel occlusions, complete internal carotid artery occlusions rarely result from them.*

What is the major learning point?

*Timely identification and appropriate management of septic embolism in high-risk populations is crucial to prevent severe neurological complications.*

How might this improve emergency medicine practice?

*Consideration of this condition in patients with known risk factors and new neurological symptoms may lead to improved long-term neurological outcomes.*

## DISCUSSION

Ischemic stroke can result from embolization of endocardial vegetations to several locations including the cerebral vasculature, spleen, kidney, and mesentery. In addition to ischemic infarctions, disseminated disease can lead to the formation of meningitis or intracerebral infection when they propagate intracranially.^5^ Typically, septic emboli are small and affect more distal branches of the cerebral vasculature. Rarely, larger vegetations embolize and occlude more substantial blood vessels such as the internal carotid artery or middle cerebral artery, resulting in debilitating neurological symptoms. Strokes and transient ischemic attacks account for between 40–50% of neurological complications of IE.^5^ Similar to strokes from a non-infectious source, the clinical presentation of a patient will depend upon the culprit vessel that is occluded.

It is important to note that despite the possibility of undiagnosed atrial fibrillation being the sole cause of this patient’s thromboembolism, his medical history and risk factors made it far more likely that his presentation was related to a septic embolism. To the best of our knowledge this is only the sixth reported case of internal carotid artery occlusions as a result of septic embolism from IE.^6^,^7^ Previous cases have been reported primarily in the context of mechanical thrombectomy retrieval.

In native cardiac valves, *S. aureus* accounts for between 30–35% of IE cases,^8^ and carries an in-hospital mortality rate between 15–30%.^9^ A multicenter prospective study conducted in 2011, which included 253 intensive care unit patients, revealed the severe prognosis of neurologic events caused by IE, with only one-third of patients surviving to discharge with functional independence.^10^

A traditional stroke evaluation including CT head without contrast and CT angiography of the head and neck should be pursued to establish the degree of ischemic insult. In the ED, TTE is recommended as the optimal modality for the initial evaluation of IE by both the American Heart Association and the European Society of Cardiology.^11^ Thrombolysis for large vessel occlusions in the setting of IE is not frequently implemented due to the high risk of hemorrhagic transformation and lower rates of favorable outcomes,^12^ and this increased risk is at least partly attributed to coexisting mycotic aneurysms.^12^ The Society of Neurointerventional Radiology recommends that mechanical thrombectomy in patients with IE who suffer a large vessel occlusion may be safe as the risks and benefits are similar to those without IE.^13^,^14^ If a patient’s clinical presentation is concerning for septic embolism, broad-spectrum antibiotics and source control are the cornerstones of treatment and should be given as soon as possible.

## CONCLUSION

We present a rare case of an internal carotid occlusion resulting from a septic embolism in the setting of infectious endocarditis. This case is unique due to the extent of the ischemic insult it caused. Septic embolism should be considered in the differential diagnosis of patients with risk factors such as IV drug use who present with new neurological symptoms. The emergency physician should consider performing a point-of-care ultrasound to look for valvular vegetations, initiating early broad-spectrum antibiotics and source control as key early steps in the management of these patients. Thrombolysis should be avoided due to the risk of hemorrhagic transformation. In certain patient populations, mechanical thrombectomy may be a possible management option.
